# Chemical sex (chemsex) in a population of French university students

**DOI:** 10.1080/19585969.2022.2042163

**Published:** 2022-06-01

**Authors:** L. Malandain, S. Mosser, S. Mouchabac, J.-V. Blanc, C. Alexandre, F. Thibaut

**Affiliations:** a University Hospital Cochin (site Tarnier), Paris University, AP-HP, Paris, France; b Hopital Saint-Antoine, Adult Psychiatry and Medical Psychology, Paris, France; c Hopital Europeen Georges Pompidou, Psychiatry, Paris, France; d INSERM U1266, Institute of Psychiatry and Neurosciences, Paris, France

**Keywords:** Chemsex, chemical sex, sexualised drugs, party and play, addiction, university students

## Abstract

**Introduction:**

Chemsex is defined by the use of psychoactive substances to facilitate or improve sexual relations. Our objectives were to assess the prevalence of the practice of ‘chemsex’ in a population of French university students and to identify socio-demographic and clinical factors associated with this practice.

**Material and methods:**

We have used an anonymous online questionnaire comprising 15 questions on socio-demographic characteristics, chemsex use, sexual satisfaction, the type of substances used in this sexual context and their route of administration.

**Results:**

A total of 680 people were included in our study. Among them, 22.5% reported chemsex behaviour in the past year. Using a multivariate analysis, factors associated with chemsex were dating application use (*p* = 0.049) and pornography use [viewing more than once per month (*p* = 0.002)]. Having a sexual partner involved in chemsex (*p* < 0.0001), celibacy (*p* = 0.007), sexual orientations other than heterosexual (*p* = 0.0013) and especially bisexuality (*p* = 0.0002) were also significantly associated with chemsex.

**Conclusion:**

This is the first study reporting a high prevalence of chemsex in a university student population. Further larger studies should be conducted to confirm these results showing a high prevalence of this at-risk behaviour.

## Introduction

‘Chemsex or chemical sex’ means the use of psychoactive substances in order to initiate, facilitate, improve and prolong sexual experiences, especially intercourse (Edmundson et al. 2018; Souleymanov et al. 2019; Guerra et al. 2020). Mostly observed in men who have sex with men (MSM; McCall et al. 2015; Sewell et al. 2017, 2019), chemsex has been associated with risky sexual behaviours as well as sexually transmitted infectious diseases (Ward et al. 2017; Pufall et al. 2018; Queiroz et al. 2019). It is most often associated with group sex practices and favoured by groups using dating apps (Bourne et al. 2014; Batisse et al. 2016; Sewell et al. 2019). In the MSM population, prevalence estimates of chemsex have ranged between 3% and 32% (Edmundson et al. 2018; Sewell et al. 2019), with four studies reporting a prevalence of 12–18% (Drückler et al. 2018; Hammoud et al. 2018; Pakianathan et al. 2018; Milhet 2019). The most frequently used substances during chemsex include crystal methamphetamine, gamma-hydroxybutyric acid/gamma-butyrolactone (GHB/GBL), alkyl nitrites (poppers) and, less often, mephedrone and other synthetic cathinones, cocaine or ketamine (Lawn et al. 2019; Maxwell et al. 2019).

Chemsex is a term most predominantly describing this practice in the MSM population. The terms ‘sexualised drugs’ or ‘party and play’ are often used more in the general population. For simplicity we have kept the term ‘chemsex‘ in our study. Very few authors have studied the prevalence of chemsex in the general population. Lawn et al. (2019) conducted a study in 22,289 people and reported a prevalence of 20% of people having used substances in order to improve sexual intercourse.

The objectives of our study were: (1) to assess the prevalence of ‘chemsex’ in a population of French university students; and (2) to analyse the socio-demographic and clinical factors associated with this sexual behaviour.

## Methods and material

### Population studied and questionnaire used

This study was conducted in Paris, France, and included a large population of university students. This study has assessed chemsex behaviour using a questionnaire including a short introduction on the objectives of the study, followed by 15 questions (Supplementary material). The questionnaire was administered anonymously via a secure internet platform, and the link to the platform was advertised in different universities in Paris. The university’s ethic committee approved the study.

The definition of chemsex used in our study was substance use in a sexual context; a past-year timeframe was set to reduce recall biases.

### Statistical analysis

Our variable of interest was the practice of chemsex considered as a binary variable (yes/no).

First, we have carried out descriptive analyses. Data were described using the usual parameters: mean ± standard deviation (S.D.) or number of subjects and percentages.

In a second step, we performed univariate analyses; Pearson’s Chi-square tests were used for categorical variables and Student’s *t* tests for quantitative variables.

Finally, we conducted multivariate analyses, using as variable of interest the use of chemsex; as adjustment variables: age, gender, marital status, level of education; and sexual orientation, the use of dating applications, pornography use, sexual satisfaction, having a sexual partner using chemsex as explanatory variables. We performed multiple linear regression analysis for continuous variables and logistic regression for qualitative variables.

Statistical significance was defined as *p* < 0.05. Analyses were performed using R software version 3.6.2 (2019-12-12).

## Results

### Characteristics of the whole sample

In total, 680 students completed the questionnaire. All questionnaires were analysed. Results are shown in Table 1.

**Table 1.** Socio demographic characteristics of the sample.

	Total population *N* = 680	Chemsex 22.5% (*n* = 153)	No chemsex 77.5% (*n* = 527)
Age (years)			
15–19	9.7% (*n* = 66)	2.8% (*n* = 19)	6.9% (*n* = 47)
20–24	6.0% (*n* = 517)	17.4% (*n* = 118)	58.7% (*n* = 399)
25–29	11.3% (*n* = 77)	2.1% (*n* = 14)	9.3% (*n* = 63)
>30	2.6% (*n* = 18)	0.3% (*n* = 2)	2.4% (*n* = 16)
Gender			
Male	24.4% (*n* = 166)	25.3% (*n* = 42)	74.7% (*n* = 124)
Female	75.3% (*n* = 512)	21.6% (*n* = 111)	78.3% (*n* = 401)
Marital status			
Single	62.6% (*n* = 426)	16.5% (*n* = 112)	6.2% (*n* = 314)
In a relationship with a partner	37.2% (*n* = 253)	6.0% (*n* = 41)	31.2% (*n* = 212)
Sexual orientation			
Heterosexual	76.6% (*n* = 521)	15.4% (*n* = 105)	61.2% (*n* = 416)
Homosexual	4.4% (*n* = 30)	0.9% (*n* = 6)	3.5% (*n* = 24)
Bisexual	14.9% (*n* = 101)	5.4% (*n* = 37)	9.4% (*n* = 64)
Pansexual	2.2% (*n* = 15)	0.7% (*n* = 5)	1.5% (*n* = 10)
Partner drug use			
Yes	24.1% (*n* = 164)	19.0% (*n* = 129)	5.2% (*n* = 35)
No	57.8% (*n* = 393)	3.5% (*n* = 24)	54.3% (*n* = 369)
Mean of frequency of pornography viewing during the last year			
Never	30.7% (*n* = 209)	4.1% (*n* = 28)	26.6% (*n* = 181)
Less than once a month	19.7% (*n* = 134)	4.7% (*n* = 32)	15.0% (*n* = 102)
Between one time per month and one time per week	26.0% (*n* = 17)	7.5% (*n* = 51)	18.5% (*n* = 126)
More than once a week	19.6% (*n* = 133)	6.2% (*n* = 42)	13.4% (*n* = 91)
Dating apps use			
Yes	17.8% (*n* = 121)	5.9% (*n* = 40)	11.9% (*n* = 81)
No	81.1% (*n* = 552)	16.6% (*n* = 113)	64.6% (*n* = 439)

### Descriptive analysis

In our sample, 22.5% of participants reported using substances to facilitate or improve sexual intercourse (chemsex practice). No significant differences in the prevalence of chemsex were observed between males and females. Almost 36.0% of them reported having used substances in a sexual context more than 5 times in the past 12 months, and about half of individuals engaging in chemsex reported using only one substance at a time (54.3%). Alcohol was the most widely used (83.7%). The results of the expected effects and the main adverse effects are shown in Table 2.

**Table 2.** Clinical characteristics of respondents engagin in chemsex.

	Chemsex use *N* = 153 (%)
Substance(s) use during sex	
Alcohol	80.4% (*n* = 123)
Cannabis	47.1% (*n* = 72)
MDMA	23.5% (*n* = 36)
Cocaine	11.8% (*n* = 18)
Hallucinogens	9.8% (*n* = 15)
Benzodiazepines	7.2% (*n* = 11)
Mephedrone and other synthetic drugs	6.4% (*n* = 10)
Poppers	5.2% (*n* = 8)
GHB/GBL	2.0% (*n* = 3)
Nitrous oxide	2.0% (*n* = 3)
Tadalafil and Vardenafil	1.3% (*n* = 2)
Opioids	0
Chemsex with partner and substance use (number of times in the past year)	
<5	64.1% (*n* = 98)
>or equal to 5	35.9% (*n* = 55)
Number of substances used	
1	54.3% (*n* = 83)
>1	45.7% (*n* = 70)
Expected effects	
Seek sexual disinhibition or ‘letting go’	83.7% (*n* = 128)
Increase sexual desire and pleasure	36.6% (*n* = 56)
Facilitate meetings	27.5% (*n* = 42)
Improve sexual performance	13.1% (*n* = 20)
Adverse consequences observed	
Forgetting condom and infectious risk (STI)	32.0% (*n* = 49)
Unwanted sex	15.7% (*n* = 24)
Unwanted pregnancy	5.9% (*n* = 9)
Loss of consciousness related to drug use	5.9% (*n* = 9)
Overdose or withdrawal symptoms	4.6% (*n* = 7)
Erection or ejaculation disorders	4.6% (*n* = 7)
Hospitalization in an emergency medical service	0
Risk of addiction	0

STI: sexually transmitted infection.

### Univariate analysis

We found a statistically significant association with chemsex in subjects declaring themselves bisexual (*p* < 0.05); in single individuals (*p* < 0.05); in those who had partners also using drugs during sex (*p* < 0.01); in those who were watching pornography more than once a month (*p* < 0.01); and in subjects using dating apps (*p* < 0.01). A significant association was also found between the search for sexual pleasure and the frequency of pornography use. No other significant associations were observed with other variables. The mean level of sexual satisfaction was not significantly different between those practicing chemsex and those not (*p* > 0.05).

### Multivariate analysis

Table 3 summarises the results of overall effects of the explanatory variables on the variable of interest; we found that chemsex was significantly associated with the use of dating applications (*p* = 0.0027), with bisexual orientation (*p* = 0.001) compared to heterosexual orientation, with a partner using chemsex (*p* < 0.0001), with being single (*p* = 0.016) and finally with the type of university training (medical study, *p* = 0.021; business study, *p* = 0.0038).

**Table 3.** Multivariate analysis of factors associated with chemsex – overall adjusted effect.

Explanatory variable	*p*
Sexual satisfaction	0.70
Dating apps use	
Yes	0.0027
Sexual orientation	
Other than heterosexual	0.001
Bisexual	0.001
Partner drug use	
Use	<0.0001
Marital status	
Single	0.016
Pornography viewing	
>Once a week	0.002

The adjusted categorical analyzes found similar results, with an overall effect of simultaneous consumption of the sexual partner, reported sexual orientation and pornography use. Table 4 summarises the results of these analyzes.

**Table 4.** Multivariate analysis of factors associated with chemsex – categorial adjusted effect.

Explanatory variable	*p*
Dating apps use	
Yes	0.046
Sexual orientation	
Other than heterosexual	0.0012
Bisexual	0.0002
Homosexual	0.69
Pansexual	0.41
Partner drug use	
Use	<0.0001
Marital status	
Single	0.007
Pornography viewing	
Between once a month and once a week	0.0008
>Once a week	0.001

## Discussion

This is the first study exploring chemsex in university students. Regarding the definition of chemsex, we used a general definition focussing on intercourse. The prevalence of chemsex reported in our population (22.5%) was the same as the high prevalence previously observed in the general population using the same definition (20%; Lawn et al. 2019). Most studies conducted in MSM populations reported similar prevalence edstimates but tended to remove alcohol and cannabis and mainly consider metamphetamine, synthetic cocaine and cathinone use (Drückler et al. 2018; Hammoud et al. 2018; Pakianathan et al. 2018). In our population, cathinones and metamphetamine were consumed infrequently, and in most cases, only one substance was used at a time; this appears to contrast with chemsex in MSM usually being associated with use of multiple drugs (Léobon et al. 2019).

The prevalence of chemsex did not differ between males and females. We found a significant association with bisexuality; a previous association was reported in a population of females having sex with females (Hibbert et al. 2019). The MSM population appears to be that practicing chemsex the most (Sewell et al. 2019; Malandain et al. 2020).

In our sample, as previously reported, frequent viewing of pornography was significantly associated with chemsex and the search for sexual pleasure (Stevens et al. 2020). The expected effects of chemsex were consistent with previous studies (Palamar et al. 2018); in MSM, the effects sought during chemsex were mainly an increase in sexual desire, arousal and pleasure (Ahmed et al. 2016; Deimel et al. 2016; Weatherburn et al. 2017; Glynn et al. 2018; Lim et al. 2018; Hibbert et al. 2019), an increase in the duration of sexual intercourse (Lim et al. 2018; Palamar et al. 2018; Hibbert et al. 2019; Choi et al. 2020), the search for disinhibition (Deimel et al. 2016; Weatherburn et al. 2017; Hammoud et al. 2018) and sexual acts that would not be practiced without substances (Ahmed et al. 2016; Deimel et al. 2016; Weatherburn et al. 2017; Glynn et al. 2018; Lim et al. 2018; Hibbert et al. 2019). We have also observed a significant association between the use of dating apps and chemsex, as reported among MSM (Drückler et al. 2018; Choi et al. 2020).

The main potential adverse effect reported by individuals engaging in chemsex was risk of infectious diseases (Batisse et al. 2018). In our study, there was also an increased likelihood of unwanted sex, which includes sexual abuse (Ward et al. 2017; Flores-Aranda et al. 2019). In addition to the prevention of sexually transmitted infectious diseases, the high prevalence of unwanted sex (15.7%) is an important factor and should be the subject of specific prevention. The risk of drug or sexual addiction, often reported among MSM, was not reported by our students, and very few reported any ‘come down phase’ symptoms (Stevens et al. 2020).

In accordance with previous studies in MSM, celibacy, having a sexual partner practicing chemsex and the use of dating apps were associated with chemsex; education level was also high in the population engaging in chemsex (Deimel et al. 2016; Hammoud et al. 2018; Lim et al. 2018).

Regarding sexual satisfaction, no significant results were observed whether individuals were involved in chemsex or not, which differs from what was has been typically reported in MSM engaging in chemsex in whom sexual satisfaction was often decreased (Hegazi et al. 2017; Léobon et al. 2019).

## Limitations of the study

Limitations of our study deserve mention: (1) the duration set at one year may have reduced the prevalence of chemsex behaviour; (2) we have used a questionnaire with several preselected answers, which may have precluded some answers; (3) females were overrepresented in our sample which may reflect a selection bias.

## Conclusion

This is the first study reporting a high prevalence of chemsex in a university student population as well as the clinical characteristics of students engaging in chemsex. Further larger studies should be conducted to confirm and extend these results.
